# Increasing ciprofloxacin resistance of isolates from infected urines of a cross-section of patients in Karachi

**DOI:** 10.1186/1756-0500-5-696

**Published:** 2012-12-27

**Authors:** Farhan Essa Abdullah, Akhtar Amin Memon, Muhammad Yasin Bandukda, Marium Jamil

**Affiliations:** 1 Department of Pathology, Sindh Medical College, Dow University of Health Sciences, Essa Lab, B-122, Block-H, Shahrah-e-Jahangir, Karachi, Pakistan; 2 Dow Medical College, Dow University of Health Sciences, Karachi, Pakistan

**Keywords:** Urine, Isolates, Ciprofloxacin, Karachi

## Abstract

**Background:**

The objective of the research was to evaluate the current effectiveness of Ciprofloxacin on the uropathogens prevalent in infected urines of a cross-section of patients in Karachi, Pakistan.

**Findings:**

An observational study conducted in a private diagnostic laboratory and its branches in key areas of Karachi City from February 2010 to July 2011. A total of 2963 consecutive urine samples were cultured on chocolate agar, CLED medium and selective EMB agar. Growth of possible uropathogens was noted, and compared retrospectively with earlier lab data of suggestive urine cultures (*n* = 1997) recorded during January 2009 and December 2009. The isolates were identified using routine procedures and the API 20 system and evaluated for their sensitivity to ciprofloxacin by Kirby-Bauer disk diffusion method. Data was subjected to statistical analysis on SPSS version 16. Out of the present-day culture-positive urines, 2409 (80.4%) yielded gram-negative rods, and 554 (18.5%) gram-positive cocci. *E.coli* (43.1%) was most frequent, followed by *Klebsiella pneumoniae* (22.4%) and *Staphylococcus aureus* (15.5%). 57.2% of the Gram-negative bacteria and 48.7% of the Gram-positive isolates were resistant to ciprofloxacin. In the earlier (2009) screening, 39% of Gram-negative rods and 48% of Gram-positive cocci were indifferent to the drug.

**Conclusions:**

A decrease in bacterial susceptibility of uropathogens to ciprofloxacin, a commonly prescribed drug in our population, is underlined, occurring possibly due to overuse pressure. Empirical initial treatment with ciprofloxacin would be inadequate in more than half of UTI cases, thereby counseling increased C/S testing of urines to provide existing sensitivity data for apt drug prescription.

## Introduction

Urinary Tract Infection (UTI) is a common finding in general clinical practice [1,2]; the primary treatment includes antibiotics as there are inadequate evidences of effective alternative therapy. Empirical antibiotic prescription is often endorsed even before the culture results are available [2], with ciprofloxacin, a synthetic 5-fluoroquinolone which functions as an inhibitor of bacterial DNA gyrase [3], a common treatment of choice for UTIs [4].

Many microbes can cause UTI. *E. coli* is the most common agent worldwide and in Pakistan [5,6] followed by Klebsiellae [7] and these uropathogens have shown a variable degree of sensitivity to ciprofloxacin [8,9]. Since the pattern of resistance may vary among them, periodic evaluation of antibacterial activity of relevant antibiotics is needed [10,11]. Also, new etiologic agents responsible for UTI have recently been identified, which also encourages the need for precise and updated population UTI surveillance data, particularly in the light of concerns regarding increasing antimicrobial resistance [7,12,13].

The pharmacodynamic and pharmacokinetic properties of ciprofloxacin initially limited its use to a last-resort therapy against infections complicated with drug-resistance [14]. However, the widespread use of this antibiotic has led to an emergence of ciprofloxacin-resistant infections, particularly when acquired in the hospital environment [15]. Clinical studies have shown that it is the pressure of drugs used in therapy that influences the resistance pattern of causative organisms more so than intrinsic bacterial protective mechanisms [16]. Hence the incapacity of physicians to prescribe tailored therapy without the knowledge of prevailing sensitivity patterns could lead to substantial morbidity and mortality in patients [17].

Accordingly, midstream urine specimens are encouraged for culturing urinary tract pathogens, and these if inoculated for example on a combination of CLED and Chocolate agars are said to increase the proficiency of lab findings [18] .Additionally, the selection of antimicrobial drugs for empiric therapy is best based on the susceptibility pattern of the species isolated in a given area and, if determined, can update the prevailing efficiency of commonly prescribed antibiotics such as ciprofloxacin.

## Findings

This study was conducted at a private diagnostic lab and its branches in key areas of Karachi City from February 1, 2010 to July 31, 2011 assessing a total of 2996 consecutive referred mid-stream urines. The actual percentage of samples the lab received from the community or local hospitals is difficult to ascertain, since specimens from hospitals are often submitted for processing by patients’ relatives without mentioning whether the patient was hospitalized or not. In general, a majority of samples are forwarded by nearby physicians and clinics, thereby reflecting the situation in the community as well as the common choice of doctors’ empirical therapy.

The samples were inoculated on enriched Chocolate agar and differential CLED and EMB media (Oxoid, UK), and which yielded significant growth of possible uropathogens after incubation for 24 hrs at 37C. Urine samples were collected from individuals who presented for the diagnostic tests of Urine DR and Urine CS.

We follow the standard routine method for labeling a urine specimen as ‘infected’ on the basis of it containing around 100,000 viable bacterial cells per ml urine gauged using a calibrated wire loop for inoculation, mostly accompanied by at least 5–10 cells per high power field in centrifuged deposit; presence of nitrite is also routinely checked using dipstick.

The isolates were speciated by routine procedures which included colonial characteristics, gram-staining, the coagulase test, API 20 system, etc. Drug sensitivity was done by the classical Kirby-Bauer Disk Diffusion Method using Sensitivity agar (Oxoid, UK) and pertinent antibiotics emphasizing ciprofloxacin with a break point of 5 μg. Although ciprofloxacin is not a drug of choice for enterococcus, it has been included in the list of antibiotics because it is among the drugs of empirical choice in Karachi.

The zone diameters were measured using calipers following methods recommended by the NCCLS standards (1987) & the WHO. It was used for determining antimicrobial susceptibility of ciprofloxacin and was confirmed by performing minimum inhibitory concentration (MIC). Resistant strains were tested for MICs wherever indicated with E-test strips.

Retrospective results recorded during January 2009 and December 2009 of a total of 1,997 suggestive urines was also evaluated for comparison; data included 1,701 Gram-negative rods (85.1%) and 216 Gram-positive cocci (14.8%).

The ethical review board of Dow University of Health Sciences approved the study.

Among the 2996 urine specimens, *Escherichia coli* (*n* = 1290) was most frequently isolated (43.1%), followed by *Klebsiella pneumoniae* (*n* = 672; 22.4%) and *Staphylococcus aureus* (*n* = 465; 15.5%). Less frequent bacteria included *Pseudomonas aeruginosa*, *Enterobacter aerogenes*, *Enterococcus fecalis, Proteus mirabilis, Streptococcus agalactiae* and occasional *Salmonella typhi*. The majority of the isolates (*n* = 2409) were Gram-negative bacteria (80.4%) itemized in Table 1. *Candida albicans* was also noted in 33 urines (1.1%) mostly accompanying MDR isolates.

**Table 1:** Percentage of urine isolates (n = 2996) during Feb 2010 and July 2011

**Gram type**	**Isolate**	**Frequency**	**Percentage**
Gram-positive n = 554 (18.5%)	Staphylococcus aureus	465	15.5%
	Enterococcus fecalis	66	2.2%
	Streptococcus agalactiae	23	0.8%
Gram-negative n = 2409 (80.4 %)	Klebsiella pneumoniae	672	22.4%
	Escherichia coli	1290	43.1%
	Pseudomonas aeruginosa	225	7.5%
	Enterobacter spp.	156	5.2%
	Proteus mirabilis	63	2.1%
	Salmonella spp.	3	0.1%

**Note:***Candida* spp. was grown in 33 of the urine samples (1.1%).

Females (*n* = 2416) constituted a major segment of the presenting individuals (80.6%), with only 19.4% males.

Infected urines were mostly excreted by the sexually active group aged 16 to 29 years (*n* = 1,001; 33.4%), and 30 to 45 years (*n* = 648; 21.6%) [p = <0.001] listed in Table 2.

**Table 2:** Frequency of isolates in infected urines of different age groups (Feb 2010 - July 2011)

**Isolate**			**Age groups**			**Total**
	**15 and below**	**16-29 years**	**30-45 years**	**46-59 years**	**60 and above**	
*Staphylococcus aureus*	18	189	113	85	60	465
*Proteus mirabilis*	2	25	6	12	18	63
*Klebsiella* spp.	24	263	159	117	109	672
*E. coli*	49	369	270	308	294	1290
*Enterococcus fecalis*	2	19	10	14	21	66
*Candida* spp.	0	12	15	5	1	33
*Pseudomonas aeruginosa*	3	72	51	43	56	225
*Enterobacter spp.*	4	41	21	43	47	156
*Streptococcus agalactiae*	1	9	2	5	6	23
*Salmonella typhi*	0	2	1	0	0	3
**Total**	**103**	**1001**	**648**	**632**	**612**	**2996**
**P-value**	**0.006**	**0.000**	**0.000**	**0.000**	**0.000**	

The susceptibility of the bacterial isolates to ciprofloxacin is presented in Table 3. 45.5% of Gram-negative rods and 51.2% of the Gram-positive isolates were found to be sensitive to ciprofloxacin. In comparison, earlier data accumulated during the 12 months of 2009 suggested that a total of 1,997 infected urines yielded 71% Gram-negatives and 52% Gram-positive cocci that were sensitive to the drug. Our current data, thus, shows a drastic decrease in sensitivity in gram-negatives, while the sensitivity of gram-positive cocci remains almost static in comparison to the previous data.

**Table 3:** Percentage sensitivity of isolates to Ciprofloxacin

**Isolates**	**Resistant (%)**	**Intermediate (%)**	**Sensitive (%)**
**Gram Negative**	**1284 (53.3%)**	**29 (1.2%)**	**1096 (45.5%)**
*Klebsiella pneumoniae*	232	13	427
*Escherichia coli*	753	09	528
*Pseudomonas aeruginosa*	135	02	88
*Enterobacter spp.*	127	03	26
*Proteus mirabilis*	37	02	24
*Salmonella spp.*	-	-	03
**Gram Positive**	**207 (37.4%)**	**63 (11.4%)**	**284 (51.2%)**
*Staphylococcus aureus*	164	50	251
*Enterococcus fecalis*	40	10	16
*Streptococcus agalactiae*	3	3	17
**Total**	**1491**	**92**	**1380**

## Discussion

Our lab-based data indicates that ciprofloxacin resistance of Gram-negative uropathogens in particular has significantly increased from 39% in 2009 to an alarming about 54% during Feb 2010 and July 2011. The antibiotic, popularly referred to as ‘Cipro’ is usually used as the drug of choice for empirical therapy in uncomplicated UTIs, even though in general it is not recommended as a first-line drug for UTI.

The emergence of resistant *E. coli* strains is the usual indication for treatment failure with drugs such as Trimethoprim-Sulfamethoxazole, and endorses shift to ciprofloxacin. Le and Miller (2001), for example, have suggested that findings of 22% resistant *E. coli* strains encourage treatment change to a Fluoroquinolone based on cost-effectiveness [19]. They also advocate that in complicated UTIs, the therapeutically significant levels of ciprofloxacin renal clearance boost its effectiveness for empirical treatment.

The widespread use of the drug has been encouraged by some studies such as those by Boerema et al. ([1985]) who reported a mere 18% resistance of isolated strains of *E. coli* and *Klebsiella *[20], and another conducted by Talan et al. in 2008 that exhibited only 5% resistance of *E.coli* treatment with ciprofloxacin in patients with complicated pyelonephritis [21].

However, in disparity, Karlowsky et al. ([2002]) conducted susceptibility tests performed for ampicillin, ciprofloxacin and nitrofurantoin, and observed that ciprofloxacin displayed a decreasing effectiveness over a period of seven years [22]. Also, Astal (2005) reported an increased rate of ciprofloxacin resistance among bacterial uropathogens emerging in Gaza strip over a period of 2 years and associated the increased rate to irrational use of the antibiotics [23]. Such studies conducted over a period of time around the globe that indicated rising levels of indifference to the drug have hopefully alerted clinicians to prescribe it prudently.

Ciprofloxacin resistance could possibly also be augmented because of its use to treat a number of maladies including UTI, gastroenteritis, infections of bones and joints, endocarditis, lower respiratory tract infection, prostatitis and particularly enteric fever, among others, in spite of its usage being associated with an increased risk of tendon rupture in all ages. Notably, perhaps contributing to the problem is its availability in oral suspension which is currently flooding our market, even though, with the exception of cystic fibrosis and inhalation anthrax, it is not licensed by the U.S. FDA for use in children due to the risk of permanent injury to the musculoskeletal system.

In keeping with established findings in literature, the majority (80.6%) of individuals presenting with UTI in our lab were females, and most of them were in the sexually active age of 16–45. Women in this age group present with UTI mostly because of their anatomical differences and behavioral patterns [24]. These infections are, however, uncomplicated, but may have been handled with popular antibiotherapy.

Males in the sexually active age group may represent those either participating in anal intercourse, or are uncircumcised, or most significantly, whose sexual partners carry uropathogens [24]. However, these reasons are not exclusively associated with the young age group, but may also account for UTI in middle-aged patients. UTI in older adults could follow bacteremia due to improper catheter management in hospitals and nursing homes [25], some or many of whom may have received antibiotics.

The age group found to have the least occurrence of UTIs is below 15 years [26] UTI in children are of significant concern and should be well assessed and treated because they may lead to malfunctioning of the urinary tract and lead to systemic abnormalities later on in life [26]. First infections are best tackled with suitable drugs rather than ciprofloxacin, not only because of its undesirable side-effects, but to reduce pressure increasing bacterial resistance.

Studies have confirmed that mortality due to UTI is not associated with age but more likely with factors such as the emergence of resistant bacteria and infections with gram positive cocci [27].

## Conclusion

UTIs are a significant patient presentation to clinicians today. The organisms isolated in our study are in accordance with the ones generally isolated over the world; however, we report increased resistance to ciprofloxacin. This may be attributable to misuse of the antibiotic, unauthorized dispensing of drugs from pharmacies, and increasing use of Fluoroquinolone in children. We recommend increased consideration of C/S testing, and intelligent empirical prescription.

## Competing interests

The authors declare that they have no competing interests.

## Authors’ contribution

AFE gave the idea of the study. AFE and MAA designed the study. MAA prepared the synopsis for IRB approval. AFE reviewed and edited the synopsis. MAA, BMY, JM collected the data. MAA and BMY entered the data on SPSS. MAA analyzed the data on SPSS. All authors wrote the manuscript. AFE reviewed and edited the manuscript. All authors read and approved the manuscript.
